# Metabolic network prediction through pairwise rational kernels

**DOI:** 10.1186/1471-2105-15-318

**Published:** 2014-09-26

**Authors:** Abiel Roche-Lima, Michael Domaratzki, Brian Fristensky

**Affiliations:** Department of Computer Science, University of Manitoba, Winnipeg, Manitoba Canada; Department of Plant Science, University of Manitoba, R3T 2N2 Winnipeg, Manitoba Canada

**Keywords:** Metabolic network, Pairwise rational kernels, Supervised network inference, Finite-state transducers, Pairwise support vector machine

## Abstract

**Background:**

Metabolic networks are represented by the set of metabolic pathways. Metabolic pathways are a series of biochemical reactions, in which the product (output) from one reaction serves as the substrate (input) to another reaction. Many pathways remain incompletely characterized. One of the major challenges of computational biology is to obtain better models of metabolic pathways. Existing models are dependent on the annotation of the genes. This propagates error accumulation when the pathways are predicted by incorrectly annotated genes. Pairwise classification methods are supervised learning methods used to classify new pair of entities. Some of these classification methods, e.g., Pairwise Support Vector Machines (SVMs), use pairwise kernels. Pairwise kernels describe similarity measures between two pairs of entities. Using pairwise kernels to handle sequence data requires long processing times and large storage. Rational kernels are kernels based on weighted finite-state transducers that represent similarity measures between sequences or automata. They have been effectively used in problems that handle large amount of sequence information such as protein essentiality, natural language processing and machine translations.

**Results:**

We create a new family of pairwise kernels using weighted finite-state transducers (called Pairwise Rational Kernel (PRK)) to predict metabolic pathways from a variety of biological data. PRKs take advantage of the simpler representations and faster algorithms of transducers. Because raw sequence data can be used, the predictor model avoids the errors introduced by incorrect gene annotations. We then developed several experiments with PRKs and Pairwise SVM to validate our methods using the metabolic network of *Saccharomyces cerevisiae*. As a result, when PRKs are used, our method executes faster in comparison with other pairwise kernels. Also, when we use PRKs combined with other simple kernels that include evolutionary information, the accuracy values have been improved, while maintaining lower construction and execution times.

**Conclusions:**

The power of using kernels is that almost any sort of data can be represented using kernels. Therefore, completely disparate types of data can be combined to add power to kernel-based machine learning methods. When we compared our proposal using PRKs with other similar kernel, the execution times were decreased, with no compromise of accuracy. We also proved that by combining PRKs with other kernels that include evolutionary information, the accuracy can also also be improved. As our proposal can use any type of sequence data, genes do not need to be properly annotated, avoiding accumulation errors because of incorrect previous annotations.

## Background

### Related work

Metabolic networks allow the modelling of molecular systems to understand the underlying biological mechanisms in a cell [1]. Metabolic networks are represented by the set of metabolic pathways. Metabolic pathways are a series of biochemical reactions, in which the product (output) from one reaction serves as the substrate (input) to another reaction. The experimental determination of metabolic networks, based on known biological data such as DNA or protein sequences, or gene expression data, is still very challenging [2]. Thus, there have been several efforts to develop supervised learning methods to determine genes coding for missing enzymes and predict unknown parts of metabolic networks [3, 4].

Most of the methods to predict metabolic networks assume that the genome annotation is correct, e.g., Pathway Tools [4], a software application to predict metabolic networks using information from BioCyc databases [5]. Pathway Tools uses a two part algorithm, in which part 1 infers the reactions catalyzed by the organism from the set of enzymes present in the annotated genome, and part 2 infers the metabolic pathways present in the organism from the reactions found in the part 1. Considering BioCyc and MetaCyc have a huge amount of available data, this application can potentially make precise metabolic pathway predictions [6]. However, part 2 is based on the annotated genes, and if there are errors in the annotation, the inferred pathways will not be correct. Therefore, these methods intrinsically carry error accumulations due to incorrect genome annotations.

To tackle this problem, we have previously proposed using information directly related to the sequence as the primary data (e.g., genomic and proteomic data) [7]. As a result, we obtained the best accuracy values using Support Vector Machine (SVM) methods combined with string kernels representing the sequence data. We experimentally demonstrated that SVMs supersede other methods, such as matrix kernel regression, for predicting metabolic networks. This is consistent with recent results showing the usefulness of SVMs in bioinformatics [8]. However, our solution [7] was computationally expensive in terms of execution time because of sequence data manipulation.

Other authors have also combined SVM and other supervised learning techniques with kernel methods to predict metabolic networks [9–11]. The main advantage of using kernel methods is that heterogeneous data can be represented and combined simultaneously. Thus, if disparate types of data can be manipulated as kernels, data from many sources can be made to contribute uniformly to the information in a training set when building a model [12].

Yamanishi [9] and Kotera et al. [11] described the theory and implementation of GENIES, a web application that allowed prediction of the unknown parts of metabolic networks using supervised graph inference and kernel methods. Several algorithms were implemented in GENIES to find the decision or predictive functions for supervised network inference. Some of these algorithms were Kernel Canonical Correlation Analysis (KCCA) [13, 14], Expectation-Maximization (EM) algorithm [15] and Kernel Matrix Regression (KMR) [9]. The authors developed several experiments, but they did not use sequence data. Therefore, one of the motivations to extend our previous research [7] was to use sequence data combined with these algorithms. As noted above, we obtained the best accuracy values with the SVM method combined with sequence kernels, but with high execution times.

To address these high computational costs, we consider the results from Allauzen et al. [16], who proposed a method to predict protein essentiality using SVMs and manipulating sequence data using rational kernels. The authors designed two sequence kernels (called general domain-based kernels), which are instances of rational kernels. To handle the large amount of data (6190 domains each with around 3000 protein sequences), automata representation was used to create the rational kernels. Their results showed that the final kernels favourably predicted protein essentiality. We note, however, that none of the previous works using rational kernels in bioinformatics [16–18] have considered problems related to biological network predictions.

Based on the fact that the rational kernels described by Allauzen et al. [16] can be extended to other problems, we define new kernels to be applied to metabolic network predictions. In this research, we represent sequence data using rational kernels. Rational kernels take advantage of the fast algorithms for, and efficient representation of, transducers for sequence manipulations to improve performance. As sequence data can be used, raw genomic or proteomic information may be considered, and this method avoids problems associated with incorrect annotation when predicting metabolic networks. Additionally, the current work is the first to combine rational kernels (using finite-state transducers) [17–20] with known pairwise kernels [10, 21–23] to obtain pairwise rational kernels. While the kernel techniques proposed in this paper can be applied equally to any machine learning tools that employ kernel methods, such as KCCA, EM or KMR, we have focused on SVMs as an illustration of their capability to reduce computational costs. We have also chosen SVM methods in light of the experimental results we obtained in previous works [7], as well as the efficiency and effectiveness of SVM methods to predict protein essentiality [16].

### Automata and transducers

Automata define a mathematical formalism to analyze and model real problems through useful machines [24]. An automaton has a set of states (generally represented by circles), and transitions (generally represented by arrows). The automaton moves from one state to another state (makes a transition) when activated by an event or function. One variant of an automaton is called finite state machine. A finite-state machine can be used to model a simple system, such as turnstiles or transit lights, or complex systems such as sophisticated spaceship controls [25].

Automata work on sequence of symbols, where *Σ*^∗^ denotes all the finite sequences using the symbols on the alphabet *Σ*, including *ε* that represents the empty symbol. In order to formally define automata and transducers, we will follow the notations used by Cortes et al. [17]. An automaton *A* is a 5-tuple (*Σ*,*Q*,*I*,*F*,*δ*) [24] where *Σ* is the input alphabet set, *Q* is the state set, *I*⊂*Q* is the subset of initial states, *F*⊂*Q* is the subset of final states, and *δ*⊆*Q*×(*Σ*∪{*ε*})×*Q* is the transition set. A transition *ι*∈*δ* describes the actions of moving from one state to another when a condition (input symbol) is encountered.

Similarly, a Finite-State Transducer (FST) is an automaton where an output label is included in each transition in addition to the input label. Based on the above definition, a FST *T* is a 6-tuple (*Σ*,*Δ*,*Q*,*I*,*F*,*δ*) [18], where the new term *Δ* is the output alphabet and the transition set *δ* is now *δ*⊆*Q*×(*Σ*∪{*ε*})×(*Δ*∪{*ε*})×*Q*. Similar to the previous definition, a transition *ι*∈*δ* is the action of moving from one state to another when the input symbol from *Σ* is encountered and the output from *Δ* is produced.

In addition, Automata and Finite-State Transducers can be weighted, where each transition is labelled with a weight. Thus, a Weighted Automaton (WA) is a 7-tuple (*Σ*,*Q*,*I*,*F*,*δ*,*λ*,*ρ*) and a Weighted Finite-State Transducer (WFST) is a 8-tuple (*Σ*,*Δ*,*Q*,*I*,*F*,*δ*,*λ*,*ρ*) [18], where the new terms *λ* and *ρ* are: , the initial weight function, and , the final weight function. The new transitions for the WAs and WFSTs are  and , respectively, where  represents the weights as real numbers.

**Figure 1:**
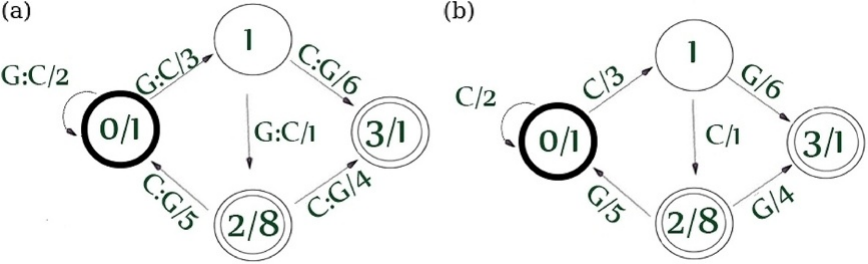
**Weighted transducer and weighted automaton representing sequences in the alphabet** ***Σ=Δ={G,C}*** **.** **(a)** Weighted Transducer *T*. **(b)** Weighted Automaton *A* (*A* is obtained projecting the output of *T*).

As an example, a weighted transducer is shown in Figure 1(a). We use as delimiters the colon to separate the input and output labels of the transitions and the slash to separate the weight values (i.e., the notation is *input:output/weight*). States are represented by circles, where the set of initial states are bold circles and the set of final states are double circles. Only the initial and final states have associated weighs (the notation is *state/weight*). Example 1 shows how to compute the weight to the transducer *T* (i.e., *T*(*x*,*y*)) for two given sequences *x* and *y*. In this case, we define the alphabets *Σ*={*G*,*C*} and *Δ*={*G*,*C*}.

#### Example 1

The weight (or value) associated to the transducer *T* in Figure 1(a) for the pair (*x*,*y*)=(*GGC*,*CCG*)∈*Σ*^∗^×*Δ*^∗^ is computed as: *T*(*GGC*,*CCG*)=1∗2∗3∗6∗1+1∗3∗1∗4∗1=48, considering that there are two accepting paths labelled with input *GCC* and output *CCG*. These paths are: *Path* 1:*State* 0↦*State* 0↦*State* 1↦*State* 3, *Path* 2:*State* 0↦*State* 1↦*State* 2↦*State* 3. The initial and final values in the terms of *T*(*GGC*,*CCG*) correspond to the weights of the initial and final states.

Figure 1(b) shows a graph representation of a weighted automaton. It can be obtained as the output projection of the transducer *T* where the input labels are omitted. Thus, the alphabet *Δ* is *Δ*={*G*,*C*} and the weight computation of the automaton *A* for two given sequences is shown in Example 2

#### Example 2.

The weight (or value) associated to the Automaton *A* in Figure 1(b) for *y*=*CCG*∈*Δ*^∗^ is computed as: *A*(*CCG*)=1∗2∗3∗6∗1+1∗3∗1∗4∗1=48considering that there are two accepting paths labelled with *CCG*. These paths are: *Path* 1:*State* 0↦*State* 0↦*State* 1↦*State* 3, *Path* 2:*State* 0↦*State* 1↦*State* 2↦*State* 3. The initial and final values in the terms of *A*(*CCG*) correspond to the weights of the initial and final states.

There are several operations defined on automata and transducers, such as *inverse* and *composition*. Given any transducer *T*, the *inverseT*^-1^ is the transducer obtained when the input and output labels are swapped for each transition. The *composition* operation of the transducers *T*_1_ and *T*_2_ with input and output alphabets both equal to *Σ* is a weighted transducer, denoted by *T*_1_∘*T*_2_, provided that the sum given by  is well defined in  for all (*x*,*y*)∈*Σ*^∗^.

#### Rational kernels

In order to manipulate sequence data, FSTs provide a simple representation as well as efficient algorithms such as composition and shortest-distance [18]. Rational Kernels, based on Finite-State Transducers, are effective for analyzing sequences with variable lengths [17].

As a formal definition, a function  is a *rational kernel* if there exists a WFST *U* such that *k* coincides with the function defined by *U*, i.e., *k*(*x*,*y*)=*U*(*x*,*y*) for all sequences *x*,*y*∈*Σ*^∗^×*Δ*^∗^ [17]. From now on, we consider the input and output alphabets with the same symbols (i.e., *Σ*=*Δ*), and only the terms *Σ* and *Σ*^∗^ will be used.

In order to compute the value of *U*(*x*,*y*) for a particular pair of sequences *x*,*y*∈*Σ*^∗^×*Σ*^∗^, the composition algorithm of weighted transducers is used [17]:

- First, *M*_*x*_, *M*_*y*_ are considered as trivial weighted transducers representing *x*, *y* respectively, where *M*_*x*_(*x*,*x*)=1 and *M*_*x*_(*v*,*w*)=0 for *v*≠*x* or *w*≠*x*. *M*_*x*_ is obtained using the linear finite automata representing *x* by augmenting each transition with an output label identical to the input label and by setting all transition, initial and final weights to one. *M*_*y*_ is obtained in a similar way by using *y*.
- Then, by definition of weighted transducer composition:
- (*M*_*x*_∘*U*∘*M*_*y*_)(*x*,*y*)=*M*_*x*_(*x*,*x*)*U*(*x*,*y*)*M*_*y*_(*y*,*y*).
- Considering *M*_*x*_(*x*,*x*)=1 and *M*_*y*_(*y*,*y*)=1, we obtain (*M*_*x*_∘*U*∘*M*_*y*_)(*x*,*y*)=*k*(*x*,*y*), i.e., the sum of the weights of all paths of *M*_*x*_∘*U*∘*M*_*y*_ is exactly *U*(*x*,*y*)=*k*(*x*,*y*).

Based on this representation, a two-step algorithm is defined by Cortes et al. [17] to obtain *k*(*x*,*y*)=*U*(*x*,*y*).


Using Algorithm 1, the overall complexity to compute one value for the rational kernel is , where |*U*| remains constant. In practice, this complexity is reduced to  in many kernels which have been used in areas such as natural language processing and computational biology. For example, Algorithm 1 for the *n*-gram kernel has a linear complexity (see a detailed description of the *n*-gram kernel below).

Kernels used in training methods for discriminant classification algorithms (e.g., SVM) need to satisfy Mercer’s condition or equivalently be Positive Definite and Symmetric - PDS [18]. Cortes et al. [18] have proven a result that gives a general method to construct a PDS rational kernel using any WFSTs.

##### Theorem 1

([18]). If *T* is an arbitrary weighted transducer, then *U*=*T*∘*T*^-1^ defines a PDS rational kernel.

#### *n*-gram kernel as a rational kernel

Hofmann et al. [26] have defined a class of similarity measures between two biological sequences as a function of the number of equal subsequences that they have. As an example of such measures is the spectrum kernel defined by Leslie et al. [27]. Similarity values are the results of summing all the products of the counts for the same subsequences. It is also referred to in computational biology as the *k*-mer or *n*-gram kernel. In the rest of this paper, we use the term *n*-gram to follow the notation of Hofmann et al. [26] and Cortes et al. [17].

The *n*-gram kernel is defined as  for a fixed integer *n*, which represents subsequences of length *n*. Here, *c*_*a*_(*b*) is the number of times that the subsequence *b* appears in *a*. *k*_*n*_ can be represented as a rational kernel using the weighted transducer , where the transducer *T*_*n*_ is defined as *T*_*n*_(*x*,*z*)=*c*_*x*_(*z*), for all *x*,*z*∈*Σ*^∗^ with |*z*|=*n* [18]. For example, for *n*=2,  is the rational kernel where *z* represents all the subsequences in *Σ*^∗^ with size 2 and *T*_2_(*x*,*z*)=*c*_*x*_(*z*) counts how many times *z* occurs in *x*.

Allauzen et al. [16] extended the construction of this kernel, *k*_*n*_, to measure the similarity between sequences represented by automata. Firstly, they define the count of a sequence *z* in a weighted automaton *A* as , where *u* ranges over the set of sequences in *Σ*^∗^ which can be represented by the automaton *A*. This equation represents the sums obtained for each *u*, of how many times *z* occurs in *u* multiplied by the weight (or value) associated to the sequence *u* in the automaton *A* (as is computed in Example 2).

Then, the similarity measure between the weighted automata *A*_1_ and *A*_2_, according to the *n*-gram kernel *k*_*n*_, is defined as:
1

Based on this definition and using Algorithm 1, the *n*-gram rational kernel can be constructed in time , as described by Allauzen et al. [16] and Mohri et al. [28].

Yu et al. [29] have verified that *n*-gram sequence kernels alone are not good enough to predict protein interactions. We address their concerns in our experiments by combining *n*-gram with other kernels that include evolutionary information.

### Pairwise kernels

We apply kernel methods to the problem of predicting relationships between two given entities, i.e., pairwise prediction. Models to solve this problem have as an input two instances, and the output is the relationship between them. Kernels used in these models need to define similarities between two arbitrary pairs of entities. Typically, the construction of pairwise kernels *K* are based on simple kernels *k*, where . In this paper four different pairwise kernels are investigated: Direct Sum Learning Pairwise Kernel [21], Tensor Learning Pairwise Kernel (or Kronecker Kernel) [22, 30, 31], Metric Learning Pairwise Kernel [23] and Cartesian Pairwise Kernel [10].

All these pairwise functions guarantee the symmetry of the pairwise kernels *K*, i.e., *K*((*x*_1_,*y*_1_),(*x*_2_,*y*_2_))=*K*((*x*_2_,*y*_2_),(*x*_1_,*y*_1_)), where *x*_1_,*x*_2_,*y*_1_,*y*_2_∈*X*. Also, if the simple kernel *k* is PDS (satisfies the Mercer condition), the resulting pairwise kernel *K* also is PDS, for each of the pairwise kernels defined above [10, 32].

#### Pairwise support vector machine

The rationale for the preceding discussion on representing disparate types of data as kernels is to enable us to use them in machine learning formalisms such as Support Vector Machines (SVMs). SVMs are used for classification and regression analysis, defined as supervised models with associated learning algorithms [33]. In this research, we use SVMs for classification. SVMs represents the data as vectors in a vector space (i.e., input or feature space). As a training set, several entities *x*_*i*_ (vectors) classified in two categories are given. A SVM is trained to find a hyperplane that separates the vector space in two parts. Each part of the feature space groups the entities into the same category. Then, a new entity *x* can be classified depending their location in the feature space related to the hyperplane [33].

Pairwise Support Vector Machines, instead, classify pair of entities (*x*,*y*) [32]. Let us formally define the binary Pairwise Support Vector Machine formulation, following Brunner et al. [32]: given a training data ((*x*_*i*_,*y*_*j*_),*d*_*i*_), where *d*_*i*_ has binary values (e.g., the pair (*x*_*i*_,*y*_*j*_) is classified as +1 or -1), *i*=1,…,*n*, *j*=1,…,*n* and the mapping function *Φ*, then the Pairwise SVM methods find the optimal hyperplane, *w*^*T*^*Φ*(*x*_*i*_,*y*_*i*_)+*b*=0, which separate the points in two categories. One of the solutions is based on the dual formalism of the optimization problem described in Cortes et al. [33]. In this case the decision function is:


where *K* is the pairwise kernel, (*x*_*i*_,*y*_*j*_) is the set of training examples, *α* is obtained from the Lagrange Multipliers as a function of *w* (the normal vector) and *b* is the offset of the hyperplane (please, see Cortes et al. [33] for more details). In this case, *α* and *b* are the “learned” parameters during the training process. Thus, *f* classifies the new pairs (*x*,*y*). For example, if *f*(*x*,*y*)> = 0, (*x*,*y*) is classified as +1, otherwise (*x*,*y*) is classified as -1.

### Metabolic networks

**Figure 2:**
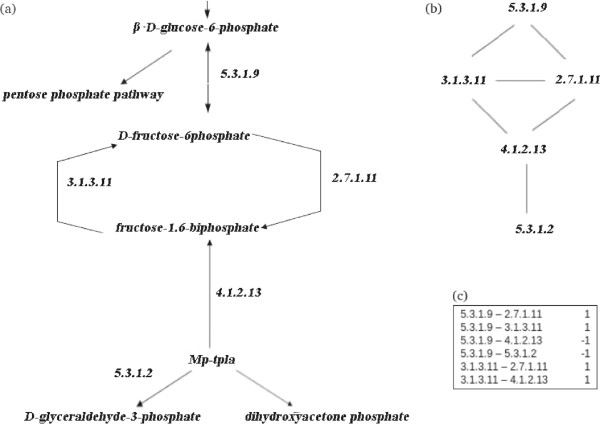
**Conversion from a metabolic network to a graph representation.** **(a)** Part of the Glycolysis Pathways, from BioCyc Database [5, 6]. **(b)** The resulting graph with the nodes (enzymes) and edges (enzyme-enzyme relations). **(c)** Table that represents known enzymes relations (EC numbers related are classified as +1 and non-related as -1).

In this work, the metabolic network is represented as a graph, in which the vertices are the enzymes, and the edges are the enzyme-enzyme relations (two proteins are enzymes that catalyze successive reactions in known pathways). Figure 2 represents a graphical transition from a metabolic pathway to a graph.

In a traditional representation of a metabolic pathway, enzymes are vertices (nodes), and metabolites are edges (branches). Following Yamanishi [9], we represent it differently, where the interactions between pairs of enzymes are considered discrete data points. For example, in Figure 2(a), the enzyme numbered EC 5.3.1.9 can create D-fructose-6-phosphate as a product, which is in turn used as a substrate by the enzyme numbered EC 2.7.1.11. This means there is an enzyme-enzyme relation between EC 5.3.1.9 and EC 2.7.1.11. Then, we create a graph in which enzyme-enzyme relations become edges and enzymes are nodes as is shown in Figure 2(b). If there is a relation between two enzymes, such a relation is classified as +1 (i.e., interacting pair). Enzyme-enzyme pairs for which no relation exists are classified as -1 (non-interacting pairs). Figure 2(c) describes these classifications, which are used as training set in the SVM method.

### Using pairwise kernel and SVM to predict metabolic networks

**Figure 3:**
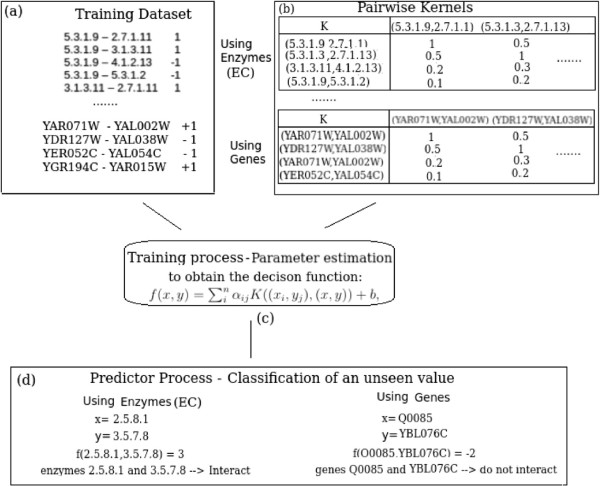
**Diagram of pairwise SVM applied to metabolic network prediction.** **(a)** An example of the pairs in the training set using the EC numbers (top) or gene names (bottom). **(b)** The pairwise kernel as a matrix, where the numerical values in each cell correspond to a measure of similarities, given two pairs of EC numbers (top) or two pairs of gene names (bottom). **(c)** A model is trained to estimate the parameters *α*_*i*,*j*_ and *b* of the decision function *f*. **(d)** Given a new pair of EC numbers (left) or gene names (right) the decision function is evaluated and the pair is classified as interacting or non-interacting.

The input data, considered as the training example dataset ((*x*_*i*_,*y*_*i*_),*d*_*i*_), is a set of known pairs of enzymes (or genes) classified in two categories (interacting or non-interacting pairs). Figure 3(a) shows an example of the input data, obtained from the metabolic network described in Figure 2(c). In Figure 3(a), enzymes are represented by EC number (top) and gene nomenclature (bottom).

Figure 3(b) represents an example of the pairwise kernel (*K*((*x*_1_,*y*_1_),(*x*_2_,*y*_2_))). Several state-of-the-art pairwise kernels were mentioned above. For example, if we consider the Tensor Product Pairwise Kernel *K* [22], then *K*((*x*_1_,*y*_1_),(*x*_2_,*y*_2_)) is computed using a simple kernel *k* (e.g., *k* could be the simple Phylogenetic (PFAM) kernel described by Ben-Hur et al. [22]). The PFAM kernel (*k*_*pfam*_(*x*,*y*)) describes similarity measures based on the PFAM database [34] between the gene *x* and the gene *y*. Thus, the Tensor Product Pairwise Kernel *K*, using as a simple kernel the PFAM Kernel *k*_*pfam*_ is defined as:


For example, in Figure 3(b)-bottom, if the genes are associated to the variables as follow: *x*_1_=YAR071W,*y*_1_=YAL002W,*x*_2_=YDR127W,*y*_2_=YAL038W, the Tensor Product Pairwise Kernel is:


A Pairwise SVM based on the dual formalism of the optimization problem is represented in Figure 3(c). The parameters *α*_*ij*_ and *b* are learned, using the pairwise kernel, *K*, and the training dataset, (*x*_*i*_,*y*_*i*_). Finally, new pairs of enzymes or genes (*x*,*y*) can be classified as interacting or not-interacting, depending the evaluation of the decision function *f* (see an example representation in Figure 3(d)). By predicting the gene interactions of the other unseen examples, all the metabolic pathways can be predicted.

The pairwise kernel computation is one of the most expensive tasks during the prediction of the metabolic networks in processing and storage. Using sequence data causes even longer execution times and large storage needs. However, we have mentioned the advantages of using sequence data in order to avoid error accumulation because of genome annotation dependencies. As well, SVMs guarantee better accuracy values than other supervised learning methods along with sequence kernels for metabolic network inference [7]. Therefore, we focus on improvement of the pairwise kernel computations and representation, by incorporating rational kernels to manipulate the sequence data. To accomplish this, we have proposed a new framework called Pairwise Rational Kernels.

## Methods

### Pairwise rational kernels

In this section, we propose new pairwise kernels based on rational kernels, i.e., Pairwise Rational Kernels (PRKs). They are obtained using rational kernels as the simple kernels *k*. We have defined four PRKs, based on the notations and definitions in the *Background* Section above.

#### Definition 1

Given *X*⊆*Σ*^∗^ and a transducer *U*, then a function  is:

- a ***Direct Sum Pairwise Rational Kernel*** (*K*_*PRKDS*_) if
- *K*((*x*_1_,*y*_1_),(*x*_2_,*y*_2_))=*U*(*x*_1_,*x*_2_)+*U*(*y*_1_,*y*_2_)+*U*(*y*_1_,*x*_2_)+*U*(*x*_1_,*y*_2_)
- a ***Tensor Product Pairwise Rational Kernel*** (*K*_*PRKT*_) if
- *K*((*x*_1_,*y*_1_),(*x*_2_,*y*_2_))=*U*(*x*_1_,*x*_2_)∗*U*(*y*_1_,*y*_2_)+*U*(*x*_1_,*y*_2_)∗*U*(*y*_1_,*x*_2_)
- a ***Metric Learning Pairwise Rational Kernel*** (*K*_*PRKM*_) if
- *K*((*x*_1_,*y*_1_), (*x*_2_,*y*_2_)) = (*U*(*x*_1_,*x*_2_)-*U*(*x*_1_,*y*_2_)-*U*(*y*_1_,*x*_2_) +*U*(*y*_1_,*y*_2_))^2^
- a ***Cartesian Pairwise Rational Kernel*** (*K*_*PRKC*_) if
- *K*((*x*_1_,*y*_1_),(*x*_2_,*y*_2_))=*U*(*x*_1_,*x*_2_)∗*δ*(*y*_1_=*y*_2_) +*δ*(*x*_1_=*x*_2_)∗*U*(*y*_1_,*y*_2_) +*U*(*x*_1_,*y*_2_)∗*δ*(*y*_1_=*x*_2_) +*δ*(*x*_1_=*y*_2_)∗*U*(*y*_1_,*x*_2_)
- where *δ*(*x*=*y*)=1 if *x*=*y* and 0 otherwise, ∀*x*,*y*∈*X*.

Following Theorem 1, if we construct *U* using a weighted transducer *T*, such as *U*=*T*∘*T*^-1^, then we guarantee *U* is a Positive Definite and Symmetric (PDS) kernel. PDS is a needed condition to use kernels in training classification algorithms. Since all the kernels defined above are results of PDS kernel operations, the PRK kernels are also PDS [35].

#### Algorithm

We have designed a general algorithm, Algorithm 2, to compute the kernels, using the composition of weighted transducers. This is a an extension of Algorithm 1. It uses as an input the transducers , , , , that represent the sequences *x*_1_,*y*_1_,*x*_2_,*y*_2_∈*X* and the Weighted Finite-State Transducer *U*, and outputs the value of *K*((*x*_1_,*y*_1_),(*x*_2_,*y*_2_)).


In our implementation described below, we use the *n*-gram rational kernel as the kernel *U* (see the *n-gram kernel as a rational kernel* Section for more details). Then, the complexity of steps (i) and (ii) are . Step (iii) adds a constant time complexity. We conclude that PRKs based on *n*-gram kernels can also be computed in time .

### Experiments

In this section we describe experiments to predict metabolic networks using pairwise SVMs combined with PRKs. We aim to prove the advantage of using PRKs to improve execution time during the computation of the pairwise kernels and the training process, while maintaining or improving accuracy values.

#### Dataset

We used data from the yeast *Saccharomyces cerevisiae* [36]. This species was selected to compare our methods, implementations and results with other methods that also predict biological networks for *Saccharomyces cerevisiae* [9, 10, 22].

The data for this species were taken from the KEGG pathway [37] and converted to a graph as described in the previous section (see Figure 2 for more details). There were 755 nodes and 2575 interacting pairs in the graph for this species. As we used SVM methods for the metabolic network inference, we prefer a balanced dataset. In this dataset, we have an unbalanced proportions of interacting (+1) and non-interacting (-1) classified pairs (e.g., for this dataset there were 282060 non-interacting pairs). In order to balance our dataset, we followed the procedure recommended by Yu et al. [29], using the program BRS-noint to select non-interacting pairs. Yu et al. [29] describes the bias towards non-interacting pair selection during the training process and the accuracy estimation. To eliminate this bias, the BRS-noint program is used to create a “balanced” negative set to maintain the right distribution of non-interacting and interacting pairs. As a result, we obtained 2574 non-interacting pairs for a total of 5149 pairs in the training process.

#### Training process and kernel computation

The known part of the metabolic network was converted in a graph and then obtained the pairs of training set, corresponding to Figure 3(a). The PRK representation coincides with Figure 3. Here, we describe the computation of PRKs (which is the main contribution of this research), given the data from the yeast *Saccharomyces cerevisiae*:

- each of the 755 known genes were represented as a trivial weighted automaton (i.e., ) using the nucleotide sequences,
- the *n*-gram kernel, with *n*=3, was used as a rational kernel, then  (see the *n-gram kernel as a rational kernel* Section for more details),
- Algorithm 2 was implemented to obtain the *K* values,
- as an example, the Tensor Product Pairwise Rational Kernel in Definition 1 is obtained by:
- *K*_*PRKT*_((*x*_1_,*y*_1_),(*x*_2_,*y*_2_))=
.
- finally, all the PRK kernels *K* with positive eigenvalues were normalized to avoid the fact that longer sequences may contain more *n*-grams, resulting in more similarities [16].

We implemented this method to compute the PRKs using Open Finite-State Transducer (OpenFST) library [38] and OpenKernel library [39]. The input data were nucleotide sequences of known genes, and the outputs were the pairwise rational kernel values as a similarity measure between pairs. Example 3 shows the input and output values for the method described above, equivalent to Figure 3(b), but using sequence data.

##### Example 3

Given nucleotide sequences *x*_1_,*y*_1_,*x*_2_,*y*_2_, which represent abbreviated examples of known genes in the dataset, *x*_1_ = GCTAAATTGGACAAATCTCAATGAAATTGTCTTGG *y*_1_ = ATGTCCTCGTCTTCGTCTACCGGGTACAGAAAA *x*_2_ = CATGACTAAAGAAACGATTCGGGTAGTTATTTGGCGG *y*_2_ = ATCTACAAGCGAACCAGAGTCTTCTGCAGGCTTAGATthe Tensor Product Pairwise Rational Kernel *K*_*PRKT*_((*x*_1_,*y*_1_),(*x*_2_,*y*_2_)) can be obtained using the 3-gram rational kernel, e.g., for *z*=*TCT*, the values are:

because, TCT appears twice in *x*_1_
- GCTAAATTGGACAAA*TCT*CAATGAAATTG
- *TCT*TGG,
because, TCT appears twice in *y*_1_
- ATGTCCTCG*TCT*TCG*TCT*ACCGGGTACAGA
- AAA,
because, TCT appears once in *x*_2_
- CATGACTAAAGAAACGAT*TCT*GGTAGTTATT
- TGGCGG, and
because, TCT appears three times in *y*_2_
- A*TCT*ACAAGCGAACCAGAG*TCT*T*TCT*GCAGG
- CTTAGAT.

With these results and other values corresponding to 3-gram rational kernel, the *K*_*PRKT*_ is computed as: *K*_*PRKT*_((*x*_1_,*y*_1_),(*x*_2_,*y*_2_))=0.3, where 0.3 is a measure of similarity.

#### SVM and predicting process

To implement the pairwise SVM method, we use the sequential minimal optimization (SMO) technique from the package LIBSVM [40] in combination with OpenKernel library [39]. During the training process, the decision function was obtained by estimating the parameters, as is shown in Figure 3(c). Now, the prediction process allows classification of new pairs of nucleotide sequences as interacting or not interacting by evaluating the decision function. Example 4 shows a description of the prediction process, similar to the process described in Figure 3(d), but using nucleotide sequences.

##### Example 4

This example describe the predictor process. Suppose we want to know if *x* = CTCAAAGTCTTAATGCTTGGACAAATTGAAATTGG, and*y*=TCTACAGAGTCGTCCTTCGTCTACCGGGAAAAT,which represent abbreviated nucleotide sequences, interact or do not interact. The decision function, *f*(*x*,*y*), was previously obtained during the training process (see the *Pairwise support vector machine* Section for more details). If the resulting value of evaluating the decision function *f*(*x*,*y*) is greater than 0, the pair (*x*,*y*) interact, otherwise the pair (*x*,*y*) do not interact. Suppose that the evaluation is *f*(*x*,*y*)=*f*(*CTCAAAGTCTTAATGCTTGGACAAATTGAAATTGG*…,*TCTACAGAGTCGTCCTTCGTCTACCGGGAAAAT*…)=+3.Then, we predict that these nucleotide sequences (*x*,*y*) interact in the context of the metabolic network of the yeast *Saccharomyces cerevisiae*. In this case, we used 755 genes during the training process, but the species has more than 6000 genes [41]. Then, the rest of the metabolic pathways can be predicted by classifying all other pairs of genes (or pairs of raw nucelotide sequences), as interacting or non-interacting, using the decision function *f*. Note that the decision function is obtained once during the training process, but can be used as often as needed during the prediction process.

The advantage of using sequence data is that nucleotide sequences can be used, even if it is not annotated. Also, any other type of sequence data, e.g., from high-throughput analysis, can be considered and combined, using a similar implementation.

#### Experiment description and performance measures

We used pairwise SVM with PRKs for metabolic network prediction, using the data and algorithms described above. We ran experiments for twelve different kernels. Firstly, we used four PRKs described in Definition 1 using the 3-gram rational kernel (i.e., *K*_*PRKDS*-3*gram*_,*K*_*PRKT*-3*gram*_,*K*_*PRKM*-3*gram*_ and *K*_*PRKC*-3*gram*_). In addition, a combination of PRKs with other kernels were considered. We included the phylogenetic kernel (*K*_*phy*_) described by Yamanishi 2010 [9] and PFAM kernel (*K*_*pfam*_) describe by Ben-Hur et al. [22]. Then, a second set of experiments were developed combining PRKs with the phylogenetic kernel (i.e., *K*_*PRKDS*-3*gram*_+*K*_*phy*_,*K*_*PRKT*-3*gram*_+*K*_*phy*_,*K*_*PRKM*-3*gram*_+*K*_*phy*_ and *K*_*PRKC*-3*gram*_+*K*_*phy*_). Finally, we combined PRKs with the PFAM kernel, obtaining *K*_*PRKDS*-3*gram*_+*K*_*pfam*_,*K*_*PRKT*-3*gram*_+*K*_*pfam*_,*K*_*PRKM*-3*gram*_+*K*_*pfam*_ and *K*_*PRKC*-3*gram*_+*K*_*pfam*_ kernels. Considering that the phylogenetic and PFAM kernels were PDS, the resulting combinations were also PDS [35].

To compare the advantages of the PRKs framework, we developed a new set of experiments with the same dataset, but without using finite-state transducers. We considered the pairwise (*n*-gram) kernel, i.e., *K*_*T*-3*gram*_. *K*_*T*-3*gram*_ denoted the pairwise tensor product described in the *Pairwise kernels* Section. To be consistent with the previous experiments, we combined the *K*_*T*-3*gram*_ kernel with the phylogenetic kernel (*K*_*phy*_) and PFAM kernel (*K*_*pfam*_), i.e., *K*_*T*-3*gram*_+*K*_*phy*_ and *K*_*T*-3*gram*_+*K*_*pfam*_ kernels, respectively. The pairwise SVM algorithm was used to predict the metabolic network using the same data set described above. Table 1 describes the groups created to compare these kernels with the equivalent PRKs.

**Table 1:** **Groups for PRK and pairwise kernel comparison**

Group	PRKs ^1^	Pairwise Kernel ^2^
N-GRAM	*K*_*PRKT*-3*gram*_	*K*_*T*-3*gram*_
PHY	*K*_*PRKT*-3*gram*_ + *K*_*phy*_	*K*_*T*-3*gram*_+*K*_*phy*_
PFAM	*K*_*PRKT*-3*gram*_ + *K*_*pfam*_	*K*_*T*-3*gram*_+*K*_*pfam*_

^1^Kernels were taken from Table 2.

^2^Computed with the Tensor Product Pairwise Kernel.

**Table 2:** **Average AUC ROC scores and processing times for various PRKs**

Exp	Type of kernels	Kernel	Average AUC score	Runtime (sec)	Confidence intervals
I	Pairwise Rational	PRK-Direct-Sum (*K*_*PRKDS*-3*gram*_)	0.499	15.0	[0.486, 0.512]
	Kernels (*PRK*)	PRK-Tensor-Product (*K*_*PRKT*-3*gram*_)	0.597	16.2	[0.589, 0.605]
	(3-gram)	PRK-Metric-Learning (*K*_*PRKM*-3*gram*_)	0.641	17.4	[0.633, 0.648]
		PRK-Cartesian (*K*_*PRKC*-3*gram*_)	0.640	15.0	[0.632, 0.647]
II	PRKs combined	PRK-Direct-Sum+Phy (*K*_*PRKDS*-3*gram*_+*K*_*phy*_)	0.425	136.2	[0.411, 0.438]
	with phylogenetic	PRK-Tensor+Phy (*K*_*PRKT*-3*gram*_ + *K*_*phy*_)	0.733	135.6	[0.725, 0.741]
	data (*K*_*phy*_ Non-	PRK-Metric+Phy (*K*_*PRKM*-3*gram*_ + *K*_*phy*_)	0.761	139.2	[0.753, 0.768]
	sequence kernel)	PRK-Cartesian+Phy (*K*_*PRKC*-3*gram*_ + *K*_*phy*_)	0.742	132.6	[0.734, 0.749]
III	PRKs combined	PRK-D-Sum+PFAM (*K*_*PRKDS*-3*gram*_ + *K*_*pfam*_)	0.493	136.2	[0.480, 0.506]
	with PFAM data	PRK-Tensor+PFAM (*K*_*PRKT*-3*gram*_ + *K*_*pfam*_)	0.827	136.8	[0.819, 0.834]
	(*K*_*pfam*_	PRK-Metric+PFAM (*K*_*PRKM*-3*gram*_ + *K*_*pfam*_)	0.844	140.4	[0.837, 0.850]
	Sequence kernel)	PRK-Cartesian+PFAM (*K*_*PRKC*-3*gram*_ + *K*_*pfam*_)	0.842	132.0	[0.835, 0.849]

All the experiments were executed on a PC intel i7CORE, 8MB RAM. To validate the model, we used the 10-fold cross validation method and measured the average Area Under the Curve of Receiver Operating Characteristic (AUC ROC) score.

Cross-validation method is a suitable approach to validate performance of predictive models. In *k*-fold cross-validation, the original dataset is randomly partitioned into *k* equal-sized subsets. Then, the model is trained *k* times. Each time, one of the *k* subsets is reserved for testing and all the remaining *k*-1 subsets are used for training. The final value is obtained as the average of the *k* results (see Kohavi et al. [42] for more details).

A Receiver Operating Characteristic (ROC) curve is a plot of the True Positive Rate (TPR) versus the False Positive Rate (FPR) for different possible cut-offs of a binary classifier system. A cut-off defines a level for discriminating positive and negative categories. ROC curve analysis is used to assess the overall discriminatory ability of the SVM binary classifiers. The area under the curve (average AUC score) has been used as a metric to evaluate the strength of the classification.

In addition, the 95% Confidence Intervals (CIs) have been computed, following the method described by Cortes and Mohri [43]. The authors provide a distribution-independent technique to compute confidence intervals for average AUC values. The variance depends on the number of positive a negative examples (2575 and 2574 in our cases) and the number of classification errors, ranging between 889 and 1912 in our cases.

## Results and discussion

Table 2 shows the SVM performance, execution times and 95% CIs grouped by the kernels mentioned above. As we can see, the experiments using only the PRK have the best execution times (Exp. I) as the transducer representations and algorithms speed up the processing. However, the accuracy is not comparable to Experiments II and III. Similar results were obtained by Yu et al. [29] with PPI networks. They stated simple sequence-based kernels, such as *n*-gram, do not properly predict-protein interactions. However, when Yu et al. [29] combined sequence kernels with other kernels that incorporate evolutionary information, the accuracy of the model predictor was improved. We obtained similar results applied to metabolic networks predictions: when the PHY and PFAM kernels were included (Experiments II and III, respectively), accuracies were improved while maintaining adequate processing times. The best accuracy value was obtained by combining the PRK-Metric-3gram and PFAM kernels (average AUC=0.844). Other papers have used similar kernel combinations to improve the prediction of biological networks, such as Ben-Hur et al. [22] and Yamanishi [9]. However, rational kernels have not been used in previous research.

Ben-Hur et al. [22] report an average AUC value of 0.78 for PFAM kernels, while Yamanishi [9] reports an average AUC of 0.77 for the PHY kernel for predicting *Saccharomyces cerevisiae* metabolic pathways. We have previously developed similar experiments but using SVM methods [7]. As a result, we obtain AUC values of 0.92 for PFAM kernel and 0.80 for PHY kernel, with execution times of 12060 and 7980 seconds, respectively. However, in all cases a random selection of negative and positive training data was used. As noted by Yu et al. [29], the average AUC values obtained by random selection of data for training machine learning tools results in a bias towards genes (or proteins) with large numbers of interactions. As such, the high AUC results in these previous works cannot be directly compared to the results in this paper. We have employed the balanced sampling techniques suggested by Yu et al. [29] to combat bias in the training set. Our results, with average AUC values in the range 0.5-0.844, are comparable to and exceed in cases the results obtained by Yu et al. [29] with balanced sampling, which range from 0.5-0.75 across several different kernels for protein interaction problems. We have also obtained these results in execution times of 15-140 seconds. With the exception of the direct sum kernel, all of the confidence intervals are above the behaviour of a random classifier.

We developed one more experiment with the PFAM kernel as a simple kernel of the Pairwise Tensor Product (*K*_*pfam*_) using a balanced sampling as suggested by Yu et al. [29]. Note that it is not a PRK; it is a regular pairwise kernel using PFAM as a simple kernel, similar to the example in the *Using pairwise kernel and SVM to predict metabolic networks* Section. As a result, the average AUC was 0.61 and the execution time was 122 seconds. When we compare these values with the results in Table 2 Exp. I, we can see that the kernels *K*_*PRKM*-3*gram*_ and *K*_*PRKC*-3*gram*_ have better average accuracy (i.e., 0.641 and 0.640, respectively) with lesser average execution times (17.4 and 15.0 seconds, respectively). In addition, when the Pairwise Rational Kernel 3-gram was combined with the PFAM kernel in the Exp. III, (i.e., Tensor Product Pairwise Rational Kernel - *K*_*PRKT*-3*gram*_ + *K*_*pfam*_), the average accuracy value (average AUC=0.827) was better than the Pairwise Tensor Product (*K*_*pfam*_), while the execution time just was increased 14.8 seconds (i.e., from 122 seconds, using *K*_*pfam*_, to 134.8 seconds, using *K*_*PRKT*-3*gram*_ + *K*_*pfam*_).

In order to statistically compares theses results, we applied the McNemar’s non-parametric statistical test [44]. McNemar’s tests have been recently used by Bostanci et al. [45] to prove significant statistical differences between classification methods. McNemar’s test defines a *z* score, calculated as:
2

where Nfs is the number of times Algorithm A failed and Algorithm B succeeded, and *N*_*sf*_ is the number of times Algorithm A succeeded and Algorithm B failed. When *z* is equal to 0, the two algorithms have similar performance. Additionally, if *N*_*fs*_ is larger than *N*_*sf*_ then Algorithm B performs better than Algorithm A, and vice versa. We computed the *z* scores considering Algorithm A as the SVM algorithm using the Pairwise Tensor Product (*K*_*pfam*_) and three different Algorithm Bs, using SVM with three different PRKs from Table 2 (i.e., *K*_*PRKM*-3*gram*_, *K*_*PRKC*-3*gram*_ and *K*_*PRKT*-3*gram*_ + *K*_*pfam*_ mentioned above). In all cases, we obtained *z* scores greater than 0 (i.e., 4.73, 4.54, 7.51), which mean the PRKs performed better. These *z*-score also proved that the difference was statistically significant with a confidence level of 99% (based on Two-tailed Prediction Confidence Levels described by [45]).

The Cartesian Kernel has not been widely used since it was defined by Kashima et al. [10]. Kashima et al. [10] used Expression, Localization, Chemical and Phylogenetic kernels to predict metabolic networks. Each of these are non-sequence kernels. In the current experiments we computed, for first time, the pairwise Cartesian kernel with a rational kernel (sequence kernel) to represent sequence data for metabolic network prediction. Cartesian kernels [10] have been defined as an alternative to improve the Tensor Product Pairwise Kernel [22] computation performance. In the three experiments shown in Table 2, we confirmed this definition, as we have obtained better accuracy and execution times when we used the Cartesian Pairwise Rational Kernel (*K*_*PRKC*-3*gram*_) rather than the Tensor Product Rational Kernel (*K*_*PRKT*-3*gram*_). Comparing our results with Kashima et al. [10], we obtained better average AUC values (i.e., 0.844 vs 0.79), and approximately the same average of the execution times (i.e., 93 seconds). Kashima et al. [10] used non-sequence data and random selection of positive and negative data for training.

**Figure 4:**
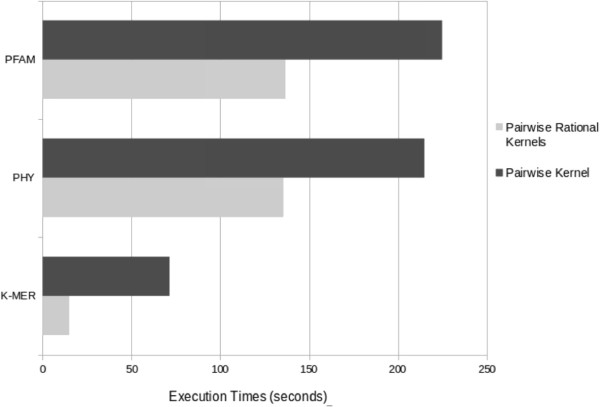
**Comparison of some pairwise rational kernels and pairwise kernels grouped by kernel types (** ***N*** **-GRAM group, PHY group and PFAM group).**

Figure 4 shows the results of the experiments comparing the PRK framework with other pairwise kernels. The three comparative groups described in Table 1 were used. As can be seen, the execution times were better when the PRKs are used in the three groups. This proves that PRKs compute faster because rational kernels use finite-state transducer operations and representations, improving the performance.

The power of using kernels is that almost any sort of data can be represented using kernels. Therefore, completely disparate types of data can be combined to add power to kernel-based machine learning methods [8]. For example, coefficients describing relative amounts of metabolites involved in a biochemical reaction (i.e., stochiometric data) can also be represented as kernels and added to strength the predicting model. For example, the reaction catalyzed by fructose-bisphosphate aldolase [EC 4.1.2.13] splits 1 molecule of fructose 1,6-bisphosphate into 2 molecules of glyceraldehyde 3-phosphate, where the relative amounts of substrate and product are represented by the coefficients 1 and 2, respectively. A stoichiometric kernel therefore would encode coefficients for all substrates and products, where enzymes that do not interact would have stoichiometric coefficients of 0. Other authors [46–48] have defined and used similar types of stochiometric data, which can be converted into kernels to be consider with PRKs.

## Conclusion

In this paper, we introduced a new framework called Pairwise Rational Kernels, where pairwise kernels are obtained based on transducer representations, i.e., rational kernels. We defined the framework, developed general algorithms and tested on the pairwise Support Vector Machine method to predict metabolic networks.

We used a dataset from the yeast *Saccharomyces cerevisiae* to validate and compare our proposal with similar models using data from the same species. We obtained better execution times than the other models, while maintaining adequate accuracy values. Therefore, PRKs improved the performance of the pairwise-SVM algorithm used in the training process of the supervised network inference methods.

In these methods, the learning process are executed once to obtain the decision function. The decision function can be used as many times as necessary to predict interaction between the other sequences in the species and predict the metabolic pathways.

The methods in this research used sequence data (e.g., nucleotide sequences) to predict these interactions. Genes do not need to be correctly annotated as the raw sequences can be used. Therefore, our methods were able to avoid the error accumulation due to wrong gene annotations.

As future work, our proposal will be used to produce a set of candidate interactions of pathways from the same and other species, that could be experimentally validated. As well, other pairwise rational kernels may be developed using other finite-state transducers operations.
